# Autophagy in inflammatory bowel disease: immunization, etiology, and therapeutic potential

**DOI:** 10.3389/fimmu.2025.1543040

**Published:** 2025-08-06

**Authors:** Zhong-Xing Miao, Huan Meng, Jie Wang, Xiao-Ting Hou, Wen-Wen Cheng, Bao-Hong Liu, Qing-Gao Zhang, Shuo Yuan

**Affiliations:** ^1^ Department of Immunology and Pathogenic Biology, Yanbian University Medical College, Yanji, China; ^2^ Chronic Diseases Research Center, Dalian University College of Medicine, Dalian, Liaoning, China; ^3^ Tangshan Gongren Hospital, Tangshan, Hebei, China; ^4^ Department of Neuroscience, Center for Brain Immunology and Glia (BIG), School of Medicine, University of Virginia, Charlottesville, VA, United States

**Keywords:** autophagy, autophagy-related genes, Crohn’s disease, inflammatory bowel disease, intestinal mucosal barrier, ulcerative colitis

## Abstract

Please confirm that the below Frontiers AI generated Alt-Text is an accurate visual description of your Figure(s). These Figure Alt-text proposals won't replace your figure captions and will not be visible on your article. If you wish to make any changes, kindly provide the exact revised Alt-Text you would like to use, ensuring that the word-count remains at approximately 100 words for best accessibility results. Further information on Alt-Text can be found here.Inflammatory bowel disease (IBD), comprising ulcerative colitis (UC) and Crohn’s disease (CD), is a chronic inflammatory disorder of the gastrointestinal tract characterized by progressive and relapsing inflammation with heterogeneous clinical manifestations. The pathogenesis of IBD involves complex interactions between intestinal barrier dysfunction and dysregulated immune responses. Autophagy, an evolutionarily conserved cellular homeostasis mechanism, plays a dual role in IBD pathogenesis by maintaining cellular integrity and modulating immune responses. This process contributes to IBD immunopathology through multiple mechanisms, including pathogen clearance, immune cell regulation, inflammatory signaling modulation, and inflammasome suppression. Growing evidence has established autophagy as a critical regulator of intestinal inflammation. Here, we described the intricate relationship between autophagy dysregulation and IBD progression, highlighting potential therapeutic strategies targeting autophagy pathways, such as inflammasome inhibitors, gut microbiota modulators, and specific signaling pathway regulators in intestinal epithelial cells and macrophages. These autophagy-focused interventions represent promising therapeutic avenues for IBD treatment. Further elucidation of the autophagy–IBD axis may provide novel insights into disease mechanisms and therapeutic development for these complex disorders.

## Graphical Abstract

Autophagy in inflammatory bowel disease: immunization, etiology, and therapeutic potential
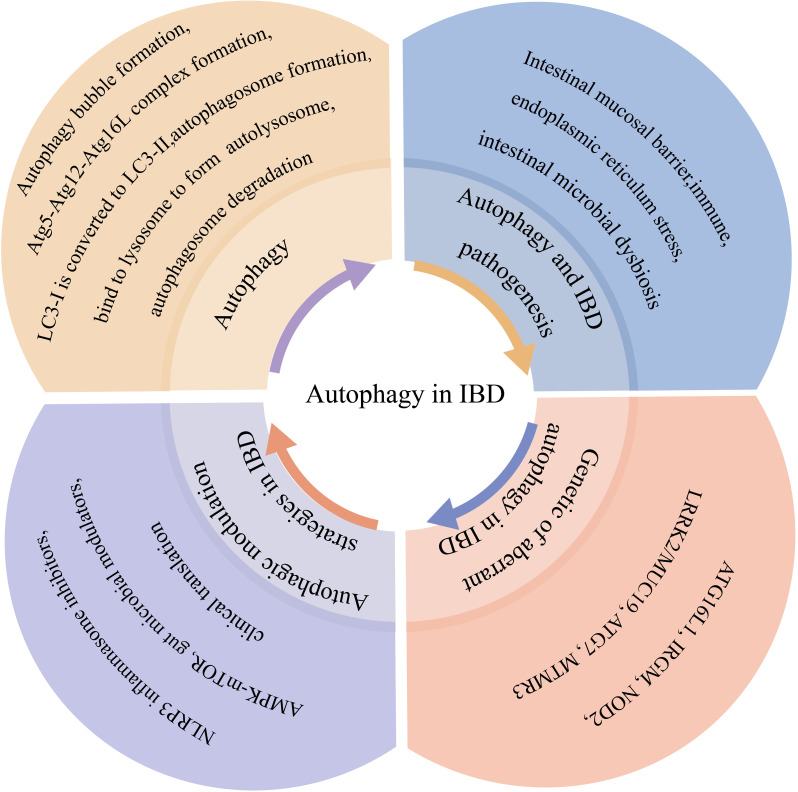

## 1 Introduction

Since 1990, the global incidence of inflammatory bowel disease (IBD) has risen markedly, with particularly pronounced increases in newly industrialized regions including Asia, Africa, South America, and the Middle East (1). IBD encompasses two main forms—ulcerative colitis (UC) and Crohn’s disease (CD)—both characterized by chronic, immune-mediated intestinal inflammation of unclear etiology. These conditions exhibit lifelong recurrence and present with debilitating symptoms including severe diarrhea, abdominal pain, intestinal inflammation, ulceration, and hematochezia (2, 3). While UC typically originates in the rectum and extends proximally, CD manifests as transmural inflammation that can affect any segment of the gastrointestinal tract (4, 5).

Current understanding positions IBD as resulting from complex interactions between genetic predisposition, environmental factors, and dysregulated host immune responses (6, 7). This triad disrupts the critical homeostasis between gut microbiota and mucosal immunity in susceptible individuals, with both innate and adaptive immune dysregulation playing pivotal roles (8). Characteristic pathological features include massive infiltration of lymphocytes and myeloid cells into intestinal lesions (9). The first-line therapies (5-aminosalicylic acid, mesalazine, azathioprine, and methotrexate) demonstrate limited efficacy and are burdened by high costs, drug resistance, dependence, and significant adverse effects (10, 11), underscoring the urgent need for novel therapeutic approaches.

Autophagy, a conserved intracellular homeostasis mechanism and regulated cell death pathway (12), has emerged as a critical player in intestinal health. Growing evidence implicates autophagy dysregulation in inflammatory diseases through multiple mechanisms, such as impaired clearance of damaged organelles and proteins, dysregulated inflammatory cytokine production, and compromised intestinal epithelial barrier function; among them, abnormal autophagy-related genes (ATGs) have a significant role (13).

This review systematically examines the association between autophagy dysfunction and IBD progression, while evaluating promising autophagy-targeting therapeutic strategies. We highlight recent advances in pharmacological modulators of autophagy and their potential to overcome current treatment limitations in IBD management.

## 2 Autophagy

In view of the central role of autophagy in IBD, we first systematically organize the molecular mechanisms of autophagy. Autophagy is a lysosome-dependent degradation pathway that eliminates dysfunctional organelles and misfolded proteins through three distinct mechanisms: macroautophagy, microautophagy, and chaperone-mediated autophagy. This catabolic process is essential for maintaining cellular homeostasis by recycling cytoplasmic components to meet metabolic demands and facilitate organelle turnover (14). Autophagy employs distinct molecular mechanisms for cytoplasmic component clearance. Thus, it can be more precisely subdivided into three types: macroautophagy, microautophagy, and molecular chaperone-mediated autophagy (15). While macroautophagy and microautophagy are evolutionarily conserved across species, chaperone-mediated autophagy is a selective process unique to mammalian cells (16). Macroautophagy (commonly referred to simply as autophagy) is characterized by the *de-novo* formation of double-membrane autophagosomes that sequester cytoplasmic cargo, which surrounds and randomly isolates cytoplasmic components before fusing with lysosomes (17).

**Figure 1:**
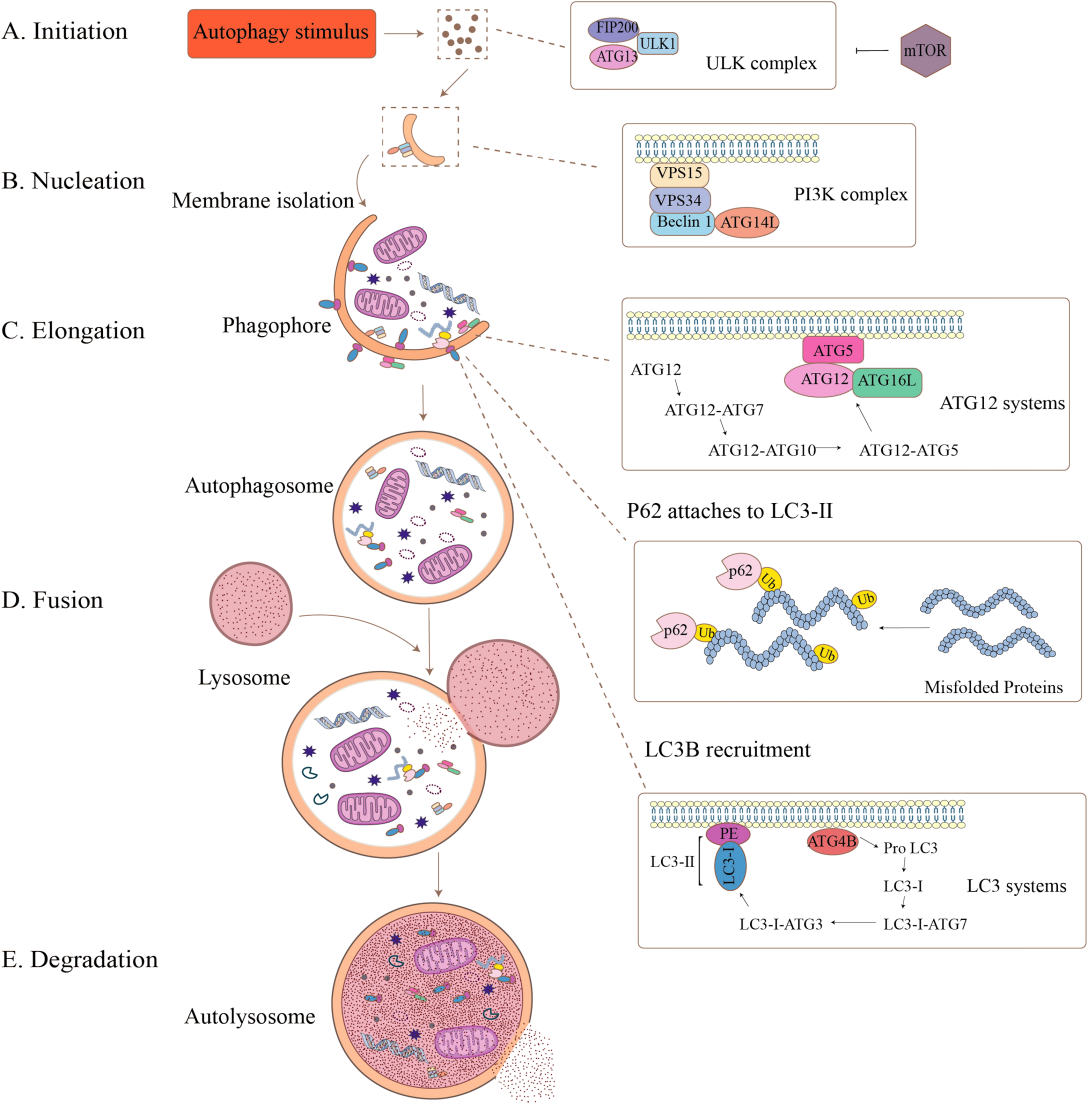
The formation process of autophagy. Autophagy is a dynamic, multistep process that can be divided into five stages: **(A)** Initiation: Autophagy-inducing stimuli activate the ULK complex (including FIP200, ULK1, ATG13), which is regulated by mTOR signaling. **(B)** Nucleation: Membrane isolation begins, driven by the PI3K complex (VPS15, VPS34, Beclin1, ATG14). **(C)** Elongation: The phagophore expands and engulfs damaged organelles and proteins, aided by the ATG12 and LC3 systems. Misfolded proteins are tagged by ubiquitin and recognized by p62, which links them to LC3-II for incorporation. **(D)** Fusion: The completed autophagosome fuses with a lysosome to form the autolysosome. **(E)** Degradation: The internal contents are degraded by lysosomal enzymes, completing the recycling process.

As shown in **Figure 1**, the autophagy cascade comprises five sequential stages: initiation, extension, autophagosome formation, autophagosome fusion, and degradation. Autophagy is initiated by the unc-51-like autophagy activating kinase 1(ULK1) complex, which phosphorylates downstream autophagic proteins, which subsequently lead to the assembly of Beclin-1, ATG14, VSP15, and VSP34 to form the class III phosphatidylinositol 3-kinase (PI3K) complex, and the ATGs recruited to the phagocytic vesicle assembly site and the isolated membrane nucleates to form a cup-like structure, also known as the phagophore (18). Membrane sources, including the endoplasmic reticulum, mitochondria, and Golgi apparatus, contribute phospholipids to expand the phagophore by donating membrane material, and the curved membrane gradually elongates and wraps into a part of the cytoplasm to form a spherical phagophore. On this phagosome, ATG16L1 forms a complex with the ATG5–ATG12 conjugate, which polymerizes microtubule-associated protein light chain3 (LC3) from a soluble form (LC3-I) to a fat-soluble form (LC3-II), and then binds to the phagophore, and the isolation membrane closes to produce bilayer vesicles to form the autophagosome (19). When most of the ATGs are cleared, the autophagosomes are sent along the microtubules to the lysosomes, where the outer membrane of the autophagosome and the lysosomal membrane fuse to form autophagic lysosomes. The monolayer within the autophagosome then releases its contents into the lumen of the lysosome, where the substances to be degraded are eventually degraded through the hydrolytic environment of the autophagic lysosome (20). During this process, some significant molecules are of high interest. LC3 and nucleoporin 62 (P62) are often used as markers of autophagic flux. The conversion of LC3-I to LC3-II indicates the closure of the phagophore, while P62 degradation indicates the fusion of autophagosomes and lysosomes (21). Extensive autophagy-related investigations have used LC3 to reflect the level of cellular autophagy. Autophagy-related genes (ATGs) and their protein products are implicated in the pathogenesis of various autoimmune disorders. Autophagy triggers autoimmune disorders, which cause various autoimmune diseases (22). Reducing the expression levels of ATGs and proteins may be a novel target for the treatment of IBD.

## 3 Autophagy and IBD pathogenesis

**Figure 2:**
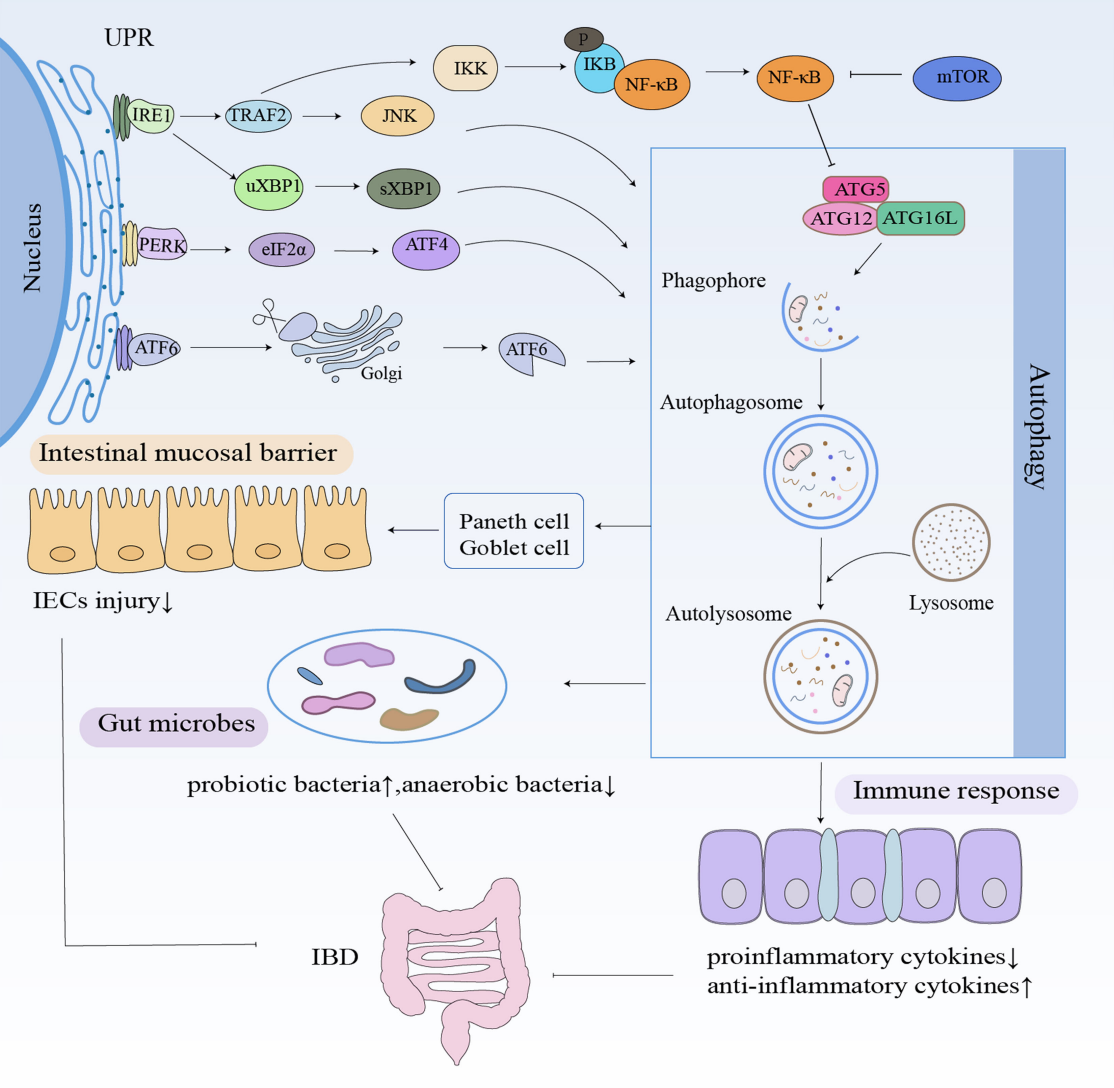
Autophagy in IBD. Autophagy contributes to IBD pathogenesis through three main mechanisms: maintaining the intestinal barrier, modulating immunity, and maintaining gut microbiota homeostasis. Autophagy stabilizes Paneth and goblet cells, increases the levels of probiotic bacterial and anti-inflammatory cytokines, and decreases the levels of anaerobic bacterial and pro-inflammatory cytokines.

As illustrated in **Figure 2**, autophagy contributes to IBD pathogenesis through three primary mechanisms: 1) intestinal barrier maintenance, 2) immune regulation, and 3) microbial homeostasis. Growing evidence demonstrates that autophagy dysfunction disrupts critical intestinal functions, thereby promoting IBD development.

### 3.1 Autophagy and intestinal mucosal barrier

The intestinal epithelium serves as the primary physical and immunological barrier against luminal pathogens, primarily through the secretion of antimicrobial peptides by specialized epithelial cells (23). The abnormal intestinal mucosal barrier function is an important basis for the pathogenesis of IBD (24). Autophagy plays a pivotal role in enhancing tight junction (TJ) integrity, notably by promoting the degradation of claudin-2 (25). The intestinal mucosal barrier is important for maintaining intestinal homeostasis. Autophagy is essential for maintaining and regulating the intestinal mucosal barrier function by regulating intestinal epithelial cell junctions, maintaining intestinal mucosal homeostasis, and participating in the repair of damage to the intestinal mucosal mechanical barrier.

Autophagy dysregulation contributes to intestinal barrier dysfunction in IBD via multiple mechanisms. The intestinal mucosal barrier is mainly composed of Paneth cells, goblet cells, absorptive cells, and enteroendocrine cells. Paneth cells regulate the balance of anti-inflammatory and pro-inflammatory factors in IBD by regulating the expression of antimicrobial peptides (26). Abnormalities in Paneth cells caused by mutations in ATGs may affect the localization of invading pathogens, bacterial recognition, antimicrobial responses, and release of antimicrobial peptides while regulating intestinal inflammatory and immune responses, which in turn affect intestinal mucosal barrier function (27). The ATG16L1 gene in Paneth cells is involved in higher levels of inflammation, but deletion of the ATG16L1 gene still leads to abnormal granule aggregation in cellular morphology and abnormal cellular granule-secreting function (28). However, it has also been shown that autophagy is specifically activated in Paneth cells from CD patients and is not associated with mucosal inflammation and disease caused by ATG16L1 or human immunity-related guanosine triphosphatase family M (IRGM) gene variants. In these Paneth cells, the activation of autophagy is mainly associated with a significant reduction in the number of secretory granules in autophagic lysosomes and their secretory autophagic profile (29). The presence of Paneth cells in the intestinal epithelium is essential for the maintenance of normal microbial homeostasis *in vivo*, mainly through autophagy to maintain normal metabolic functions of the host (30). However, when Paneth cells become dysfunctional due to environmental or genetic factors, antimicrobial peptides, for example, are not secreted properly, leading to differential dysbiosis in the composition of the normal intestinal flora, namely, intestinal disorders, which is an important cause of IBD. In CD patients with allogeneic autophagy disorders, invasion of intestinal epithelial cells by adherent invasive *Escherichia coli* or *Salmonella typhimurium* is due to autophagic dysfunction of Paneth cells (31). Autophagy also controls the secretory function of goblet cells. The nucleotide-binding oligomerization domain, leucine-rich repeat and pyrin domain-containing (NLRP) inflammasome, promotes cellular autophagy in NLRP6-deficient mice by promoting goblet cell mucin granule cytosolic exocytosis. The goblet cells will not be able to secrete mucin efficiently after damaged autophagy, and thus, the epithelial barrier will be impaired (32). The studies demonstrate that the relationship between autophagy and the intestinal mucosal barrier may be related to the development of IBD.

### 3.2 Autophagy in immune cells

Autophagy performs different functions in different immune cells. The overexpression or underexpression of this mechanism can have variable effects depending on the cell type.

Neutrophils are essential characters in the resistance to bacterial and fungal infections, and neutrophil-mediated secretion of the pro-inflammatory cytokine IL-1β was shown to be significantly reduced in bone marrow-specific ATG5- and ATG7-deficient murine models (33). The knockdown of the autophagy protein ATG5 or the use of autophagy inhibitors can effectively reduce inflammation (34). Autophagy is widely utilized in dendritic cells (DCs). In DCs derived from peripheral blood mononuclear cells, suppression of autophagy shows a decrease in interleukin (IL)-10 levels, inducing more proliferation of T cells (35). The regulatory T cells (Treg cells) inhibit autophagy in DCs in a cytotoxic T lymphocyte-associated protein 4-dependent (CTLA4-dependent) manner, thereby effectively suppressing inflammatory response-mediated autoimmune disorders (36).

Metabolism and autophagy are critical for T cells that form the basis of adaptive immunity in inflammatory diseases, which also provide possible therapeutic targets. The knockdown of ATG5 from CD8^+^ T cells displays more effector memory phenotype T cells, produces high levels of interferon-γ (IFN-γ) and tumor necrosis factor α (TNF-α) (37), exhibits an overreaction to antigens and exacerbation of symptoms, and contributes to the development of more disease. Treg cells play a key role in maintaining immune tolerance. The Treg cell population has a dual function, and the inflammatory environment promotes Treg cells toward a pathogenic phenotype (38). CD4^+^ T cells orchestrate adaptive immune responses by producing cytokines and effector molecules. Autophagy is an important process that regulates CD4^+^ T-cell function. Through lysosomal degradation of cytoplasmic material, autophagy mediates CD4^+^ T-cell-mediated immune responses, including cytokine production, proliferation, and differentiation. In addition, through a canonical process involving the autophagic machinery, autophagy contributes to the development of chronic inflammatory diseases, and autophagy-mediated CD4^+^ Treg cells are promising therapeutic targets for the treatment of inflammatory diseases (39).

Autophagy is differentially activated in B cells. Deletion of the autophagy gene ATG7 leads to failure of B-cell self-renewal and accumulation of dysfunctional mitochondria, which fail to maintain basic biological functions (40). The differentiation of B cells into plasma cells requires the involvement of autophagy, and the endoplasmic reticulum stress produced by plasma cells during immunoglobulin secretion renders them extremely dependent on autophagy regulation. Interestingly, B cells generate different cellular responses in response to different stimuli, prompting a transient switch from typical to atypical autophagy, which is essential for controlling B-cell differentiation and fate (41).

Autophagy, as a major pathway, serves an essential function in immune cell function. It is important clinically to understand the changes in autophagy in specific cell types during these phases. Ideally, autophagy-modulated therapies should consider the dynamics of cell specificity and disease mechanisms. In order to obtain promising cell-specific drugs, it is important to study the regulatory mechanisms. Clarifying the role of autophagy in immune cells will provide effective guidance for the treatment of IBD patients.

### 3.3 Autophagy in immune response

Autophagy is broadly involved in the growth, metabolism, and immune defense functions of the body, which is inextricably linked to IBD. Autophagy may be involved in the occurrence and development of IBD by multiple mechanisms (42).

Autophagy can control the adaptive immune response by participating in the antigen presentation process. The major histocompatibility complex (MHC)-II can stimulate lymphocytes through the presentation of processed antigens, triggering an adaptive immune response. Autophagy transports a variety of antigenic substances into the lysosome and constitutes an essential mechanism for the origin of MHC-II class antigens. In addition, persistent bacterial infection may be the etiology of IBD. Adherent-invasive *Escherichia coli* (AIEC) infection is often present in the intestinal epithelial cells of CD patients with ileal involvement. AIEC inhibits ATG16L1 expression and thus autophagy by downregulating the nuclear factor kappa-B (NF-κB) pathway. The downregulation of autophagy leads to increased intracellular infection, recruitment of more inflammatory cell infiltration, secretion of more pro-inflammatory factors, and intensification of CD development (43).

It was found that physiological levels of autophagy effectively inhibited AIEC proliferation, whereas ATG16L1 and IRGM gene-deficient cells had significant AIEC proliferation. Defective autophagy leads to reduced intracellular bacterial clearance, perpetuates intracellular infection, recruits more inflammatory cell infiltration, and causes cytokine overproduction and the formation of chronic granulomas, all effects that may be associated with the IBD pathogenesis (44). The expression of IRGM can be significantly induced by CD-associated AIEC monocyte infection, lipopolysaccharide (LPS), or cytosolic acyl dipeptide (45). Once host cells can inhibit the intracellular multiplication of bacteria by inducing autophagy, AIEC invades and abnormally colonizes the intestinal mucosa of patients with CD, thereby causing an inflammatory response.

In both immune homeostasis and the regulation of the inflammatory response, NF-κB has a significant function (46). The NF-κB pathway is activated by the receptor protein BCL10, a selective autophagy that regulates T-cell receptors, and when T cells are activated in the inflammatory response, autophagy can selectively restrain the formation of the bridging protein BCL10 complex to decrease NF-κB activity, thus preventing its overactivation (47). It has been indicated that the mammalian target of rapamycin (mTOR), an inhibitory-like pathway in autophagy, is activated in IBD animals and inhibits autophagy in intestinal epithelial cells through the NF-κB pathway to regulate intestinal inflammation (48). The NF-κB signaling pathway, which regulates inflammatory cytokines, is a key mechanism and an important therapeutic target for CD development (49). The adoption of interventions in the NF-κB signaling pathway to further modulate autophagy mechanisms can not only bring new ideas for the diagnosis of IBD but also facilitate the development of new therapeutic opportunities for IBD.

Autophagy can inhibit inflammasome activation by degrading inflammasome components and intracellular residual waste. The inflammasome is a multiprotein complex assembled by intracytoplasmic pattern recognition receptors (PRRs) and is an integral part of the natural immune system. The inflammasome recognizes pathogen-associated molecular patterns (PAMPs) or host-derived danger signaling molecules and damage-associated molecular patterns (DAMPs) that recruit and activate the pro-inflammatory protease caspase-1. The activated caspase-1 cleaves the precursors of IL-1β and IL-18, producing the corresponding mature cytokines and promoting the inflammatory response process in the body (50). Autophagy prevents the activation of the inflammasome by these endogenous inflammatory agents by degrading intracellular residual waste such as abnormal protein aggregates and aging-defective organelles. Not only that, but autophagy also inhibits its activation by degrading inflammasome components.

Autophagy, as a cellular self-protection mechanism, performs a complex and important function in immune regulation and inflammatory response in the gut. Different types of immune cells are affected by autophagy, and dysregulation of autophagy may lead to exacerbated inflammation or imbalanced immune function. This aspect suggests a dual role for autophagy in gut health, i.e., it both protects the host from infection and may promote inflammation in some cases. Therefore, understanding the specific mechanisms of autophagy’s role in these cells is crucial for the development of new therapeutic targets. However, the complexity of studying the role of autophagy in the gut also makes it important to be cautious of conflicting results from different studies. Some studies suggest that enhanced autophagy may help reduce inflammation, while others point out that overactive autophagy may lead to immune dysregulation. Therefore, future studies need to delve deeper into the balance of autophagy regulation and how this balance is achieved in different pathological states.

### 3.4 Autophagy and endoplasmic reticulum stress

The endoplasmic reticulum is an important organelle in the cell associated with protein folding, modification, transport, and secretion functions (51). The normal function of the endoplasmic reticulum can be affected in response to stimulation by a variety of stressors, resulting in unfolded or misfolded proteins, a process known as endoplasmic reticulum stress (52). Endoplasmic reticulum stress can couple to intracellular inflammatory response signaling pathways and is a major cause of inflammatory responses triggered by non-infectious pathogens. The unfolded protein response (UPR) is mediated through the protein kinase R-like endoplasmic reticulum kinase (PERK), inositol-requiring enzyme (IRE)-1, and activating transcription factor (ATF)-6, three signaling pathways that stimulate inflammatory responses, which in turn activate the NF-κB signaling pathway. Intestinal endoplasmic reticulum stress can lead to abnormal Paneth cell secretion function, which impairs the normal intestinal antimicrobial and mucin barriers, which in turn causes sustained inflammatory stimulation and promotes the development of IBD (53). In response to endoplasmic reticulum stress, cells can restore endoplasmic reticulum homeostasis by removing misfolded proteins and activating unfolded protein responses. Autophagy has a major contribution to maintaining cellular homeostasis by removing unfolded proteins, protein aggregates, and broken organelles. The increased endoplasmic reticulum stress and enhanced NF-κB and TNF signaling pathways in autophagy-deficient mice can cause spontaneous ileitis similar to CD manifestations (54). The above findings suggest that autophagy and endoplasmic reticulum stress may have a close relationship with IBD development.

### 3.5 Autophagy and intestinal microbial dysbiosis

The gut microbiota relies on a large microecosystem that also performs nutrient uptake and defense functions. The gut contains 1,000–5,000 different species, where 99% are from the phyla *Firmicutes*, *Bacteroidetes*, *Proteobacteria*, and *Actinobacteria* (55). The collective genome of these microbes encodes roughly 100 times more genes than the human genome. Coexistence with flora favors host metabolism and gastrointestinal tract development (56). Symbiotic microorganisms can protect the host from intestinal pathogenic infections by colonizing resistance and synthesizing factors that promote reciprocal symbiosis, and alterations in the bacterial flora can significantly contribute to the development of IBD. An increased number of mucosal bacteria, a decreased number of anti-inflammatory commensal *Clostridium perfringens*, and an increased number of paratuberculosis and adherent-invasive *E. coli* and *Clostridium difficile* were found in the intestines of CD patients (57). In patients with CD, it was also found that abnormal changes in gut microbes can expand the inflammatory response, attracting a large number of infiltrating T cells, B cells, macrophages, dendritic cells, neutrophils, and so on to the lamina propria, while the body’s autoimmune response is unable to exert a defensive role in order to control intestinal inflammation, resulting in the production of a large number of pro-inflammatory factors in local tissues by the activated lamina propria cells, including TNF, IL-1β, and others (58).

The normal intestinal flora constitutes the biological barrier of the intestinal mucosa, and the dynamic balance of microorganisms plays a pivotal role in the course of chronic IBD. It was found that autophagy plays a major function in protecting cells from *Salmonella* and *Shigella*, as well as preventing the spread and dissemination of bacteria to other organs (59). The intestinal epithelial cells are the first line of defense against bacterial invasion, which prevents contact between bacteria and the intestinal epithelium by secreting antimicrobial proteins (60). However, bacteria such as *Salmonella* or certain opportunistic commensal bacteria can leap over the first line of defense to invade the intestinal epithelium (61). Autophagy can recognize and degrade these pathogenic bacteria (62). Conditionally pathogenic bacteria such as *Enterococcus* have been reported to cause autophagy in small intestinal epithelial cells, with their target often being autophagosomes, further demonstrating that conditionally pathogenic bacteria are a prerequisite for autophagy in intestinal epithelial cells (63).

In IBD patients, probiotic bacteria are significantly decreased, while anaerobic bacteria are markedly increased. In particular, an overgrowth of AIEC has been shown to compromise intestinal permeability, disrupt the diversity and composition of the gut microbiota, and promote inflammation by modulating the expression of inflammatory genes, thereby contributing to intestinal inflammation (64). For dextran sulfate sodium (DSS)-induced colitis mice, the gut microbiota composition of ATG16L1^T300A^ knockout mice showed a decrease in *Sclerotinia phylum* and an increase in *Bacteroidetes*, *Proteobacteria*, and *Cyanobacteria* compared to normal mice (65). The mechanism by which autophagy regulates the gut microbiota and thus affects the development of IBD is not clear and needs further study, but it also provides a new direction for the treatment of IBD.

## 4 Genetic variants related to inflammatory bowel disease and autophagy

Autophagy, regulated by evolutionarily conserved ATG, serves a fundamental function in the dynamic homeostasis of cells, tissues, and organisms, in which a clear etiological link exists between mutations in the genes controlling autophagy and human diseases. It has been demonstrated that autophagy is associated with susceptibility to CD, and the pioneering evidence for the involvement of autophagy in the etiology of IBD comes from a genome-wide association study (GWAS) (66), where ATG has an important role in protecting the intestinal mucosal barrier from infection and inflammatory challenges. Among the many CD-related risk genes, ATG16L1, IRGM, nucleotide-binding oligomerization domain 2 (NOD2), protein tyrosine phosphatase non-receptor type 2 (PTPN2), leucine-rich repeat kinase 2 (LRRK2), and transcription factor EB (TFEB) have essential roles in maintaining colonic immune homeostasis and correlate highly with defective autophagy in immune cells and intestinal epithelial cells, providing strong support for autophagy in the development of IBD (**Table 1**) (98).

**Table 1:** Genetic variants related to inflammatory bowel disease and autophagy.

Gene	Relation to autophagy	Association with IBD	Reference
ATG16L	Participates in autophagosome assembly and forms a complex with the ATG5–ATG12 conjugate	Anti-inflammatory effects and regulate macrophage function and gut microbiota balance	Parzych and Klionsky (19) Zhang et al. (67) Iida et al. (68) Pott et al. (69) Gammoh (70) Quiroz-Cruz et al. (71) Shao et al. (72)
IRGM	Autophagy activation and phagocytic vesicle maturation; mediates P62-dependent autophagic degradation	Regulates NLRP3 by mediating its SQSTM1/P62-dependent autophagy, decreases pro-inflammatory factors, and inhibits pathogenic bacteria	Liu et al. (73) Mehto et al. (74) Mehto et al. (75) Finethy et al. (76) Larabi et al. (77) Aonuma et al. (78), Tao et al. (79)
NOD2	Targets ATG16L1 and recruits the autophagy protein ATG16L1 to the plasma membrane at the bacterial entry site	Bacterial sensing, miRNA-mediated post-transcriptional regulation; affects sIgA transport and activates the NF-κB and MAPK pathways	Travassos et al. (80) Marchiando et al. (81), Salem et al. (82), Zhou et al. (83) Carlos et al. (84) Dowdell and Colgan (85)
LRRK2/MUC19	The interaction with Beclin-1 leads to the phosphorylation of Beclin-1	Regulates the production of inflammatory cytokines, activation of transcription factor NFAT, and reactive oxygen	Gardet et al. (86) Liu et al. (87) Zhang et al. (88) Takagawa et al. (89)
ATG7	Promotes the formation of autophagic vesicles	Regulates microbial composition, antimicrobial peptide expression, and colonic mucin secretion and is involved in the compensatory process of intestinal epithelium during ongoing endoplasmic reticulum stress	Fujishima et al. (90), Adolph et al. (91), Nighot et al. (25) Tsuboi et al. (92) Ravindran et al. (93)
MTMR3	Modulates the local PtdIns3P levels and negatively regulates autophagy	Regulates intestinal immune homeostasis	Taguchi-Atarashi et al. (94) Lahiri et al. (95) Senousy et al. (96)
HMGB1	Protects Beclin-1 and ATG5	Reduces apoptosis and maintains tissue barrier integrity	Zhu et al. (97)

### 4.1 ATG16L1

The ATG16L1 gene is a protein involved in encoding a metabolic pathway that deals with autophagosomes, and genetic variants in ATG16L1 are associated with the risk of IBD development in different races (99). ATG16L1 is a homolog of ATG16 and is essential in the formation of autophagosomes. A partial single nucleotide polymorphism (SNP) in the ATG16L1 gene was found to be strongly associated with the development of CD by GWAS, with the most common SNP-associated locus being rs2241880 in exon 9 (100). It was demonstrated in ATG16L1-deficient mice and isolated organoid research using small intestinal epithelial cells that ATG16L1 reduces inflammation-induced apoptosis, maintains barrier integrity, and alleviates chronic intestinal inflammation (69). Enhanced expression of pro-inflammatory factors in IBD patients is thought to be related to the malfunction of autophagy and its associated genes (72). ATG16L1 is also important in myeloid cells. The TIR structural domain-containing adaptor-inducing interferon-β (TRIF)-dependent inflammasome activation occurs in macrophages lacking ATG16L1, which in turn produces large amounts of inflammatory cytokines IL-1β and IL-18. Deficiency of ATG16L1 in hematopoietic cells resulted in increased susceptibility to DSS-induced colitis, suggesting a critical function for ATG16L1 in the regulation of intestinal inflammation. Studies in mice with myeloid ATG16L1 deficiency have shown that ATG16L1 deficiency leads to an increased inflammatory response as well as an increase in colitis-causing bacteria, suggesting that ATG16L1 deficiency leads to altered macrophage function, which exacerbates CD (67).

The ATG16L1 gene is prone to mutations, such as the mutation of codon 300 threonine to alanine on the 47th gene of ATG16L1. A mutated ATG16L1 gene leads to a decrease in its intracellular expression, which results in enhanced expression of monocyte inflammatory factors induced by bacterial metabolites. This situation has been somewhat confirmed in studies targeting other diseases. Targeting the role of autophagy concerning lung infection, it was found that mice defective in the ATG16L1 gene have increased mortality after infection with *Chlamydia pneumoniae*, which may be associated with defective autophagy leading to inflammasome activation and increased production of the cellular inflammatory factor IL-1β (101). This finding also indicates, to some extent, that the ATG16L1 gene has an anti-inflammatory effect. Furthermore, the endoplasmic reticulum stress sensor inositol-requiring enzyme-1α (IRE1α) was accumulated in Paneth cells of Atg16l1^ΔIEC^ mice, and ATG16L1^T300A^ homozygotes showed a corresponding increase in IRE1α in intestinal epithelial crypts, resulting in intestinal inflammation (102). The loss of IKKα function significantly impairs the secretion of cytoprotective IL-18 and upregulates the endoplasmic reticulum stress response by reducing the stability of ATG16L1 (103).

Most of the evidence for an association between ATG and IBD etiology comes from functional studies using the ATG16L1^T300^ variant, a pivotal protein in autophagic vesicle formation, and its correct localization is critical for autophagosome formation (104). Evaluation of the function of ATG16L1^T300A^ polymorphism heterozygotes and pure heterozygotes in knock-in mice revealed that both types of KI mice exhibited defective macrophage autophagy induction. Thus, both types of mice exhibited defective bacterial clearance accompanied by an increase in the inflammatory cytokine IL-1β. Also in the study, macrophages from both types of KI mice were found to be defective in TNF-α-induced ATG16L1^T300A^ cleavage, increased bacterial retention, bacterial transmission, and *Salmonella*-induced colitis (105). One of the most prominent functions of autophagy in immunity is to inhibit the production of cytokines involved in host defense, including IL-1β and interferon (IFN)-I, giving autophagy an important role in controlling the immune system and inflammatory response in the intestinal mucosa (106). The abnormal ATG16L1 gene leads to autophagy dysfunction, causing increased production of inflammatory cytokines by innate immune cells and abnormal function of intestinal epithelial cells, resulting in an abnormal immune response in the gut that is closely related to the pathogenesis of IBD (68).

In recent years, a novel autophagy regulator microRNA 223 (miR223) targeting the ATG16L1 gene has been identified. It was found that if the ATG16L1 gene is mutated, it disrupts the action of miR223, which in turn increases or limits the uncontrolled and potentially harmful autophagic activity in cells (107). When the level of the ATG16L1 gene is reduced, it leads to abnormal autophagic activity of the body, disrupting the microbial homeostasis of the body’s gut and leading to increased secretion of pro-inflammatory factors (108). Beyond that, cells defective in the ATG16L1 gene cause a block in the recognition and presentation of bacterial antigens by NOD2, which prevents the normal initiation of the adaptive immune response (71). On the other hand, the ATG16L1 gene also performs an active role in the formation of autophagosomes (70). When the ATG16L1 gene is missing or mutated in IBD patients, the autophagy of the patient’s organism will be disrupted, while the autophagy level of the cells will be significantly reduced, and it is even impossible to form autophagosomes. Such defective autophagy cells caused by ATG16L1 gene deletion or mutation cannot effectively degrade the microorganisms engulfed in the cytoplasm, and bacterial antigens cannot be presented to T cells, so the body cannot effectively remove harmful factors and promote the development of inflammation. In conclusion, in the development of IBD, the normally expressed ATG16L1 gene can perform certain anti-inflammatory effects. In contrast, the mutation or deletion of the ATG16L1 gene can exacerbate IBD by promoting the release of inflammatory factors, blocking antigen presentation, and inhibiting autophagosome formation.

### 4.2 IRGM

The immunity-related GTPase (IRG) gene family is associated with anti-infective and inflammatory immune processes, of which only two gene types, IRGC and IRGM, have been detected in humans. The IRGM gene is a major negative regulator of the IFN response, maintaining IFN homeostasis, interacting with nucleic acid sensor proteins, and mediating P62-dependent autophagic degradation, thereby inhibiting IFN signaling (109). If the IRGM gene is defective, a strong IFN response is induced. A strong association between IRGM gene variants and CD susceptibility has been found, especially SNPs (rs10065172 and rs13361189) that can increase the risk of clinical subphenotypes of CD (fibrous contractile behavior, peritoneal disease, etc.) (110).

IRGM1 can target *Mycobacterium* phagocytosis microsomes through lipid-mediated interactions to enhance phagocytosis maturation and antimicrobial effect (111). IRGM can regulate autophagy by translocating to the mitochondria, thereby affecting mitochondrial fission, which is required for autophagic defense against intracellular mycobacteria. IRGM was found to be induced by IFN-γ in IRG-deficient mice and to play a role in the clearance of bacteria such as *Toxoplasma gondii*, *Listeria monocytogenes*, and *Salmonella*. During infection, human IRGM and murine IRGM1 activate autophagy by recruiting autophagy and SNARE (N-ethylmaleimide-sensitive factor adaptor protein receptors) bridging proteins to promote cell-autonomous defense (112). However, IRGM supports viral replication through autophagy activation, such as IRGM translocation to the Golgi apparatus, where it regulates Golgi membrane breakage and participates in virus-triggered autophagy activation during hepatitis C infection (113).

IRGM has a protective role in the maintenance of intestinal homeostasis. *Irgm1*-deficient mice exhibit defective intestinal Paneth cell function and excessive inflammatory responses in the colon and ileum after chemical exposure (73). IRGM/Irgm1 negatively regulates NLRP3 by inhibiting its activation to de-suppress the inflammatory response induced by pathogenic stimuli, thus providing some protection to the intestine and achieving a preventive effect against inflammation (75). The IRGM gene also negatively regulates NLRP3 by mediating its SQSTM1/P62-dependent autophagy (74, 78). The IRGM gene is a negative regulator of caspase-11 activation in macrophages (76). When macrophages are exposed to extracellular LPS, bacterial outer membrane vesicles, or Gram-negative bacteria, macrophages lacking IRGM2 expression aberrantly activate caspase-11-dependent inflammatory responses. In addition, miRNA-mediated post-transcriptional regulation serves an essential role in autophagy associated with IBD, whereby when autophagy genes ATG16L1 and IRGM are mutated, AIEC inhibits the autophagic defense process by increasing the levels of miR-30c and miR-130a to suppress the expression of normal autophagic proteins such as ATG16L1 in host cells (77). Reduced IRGM gene expression increases pro-inflammatory factors (114). It was found that mice with IRGM gene deletion had increased susceptibility to non-invasive intestinal pathogens, were unable to control their growth in the intestine, and showed impaired autophagy, which in turn promoted intestinal inflammation and even led to host death (115). Thus, the IRGM gene itself influences the inflammatory response in IBD to some extent. However, there are relatively few relevant studies on the mechanism of IRGM gene action in UC, which can be a direction for future research.

IRGM proteins inhibit CD-related intracellular AIEC in epithelial cells through autophagy activation and phagocytic vesicle maturation. More pathogenic AIEC was found in the intestinal mucosa of CD patients than in healthy controls, where it invaded intestinal epithelial cells and induced TNF-α production. This suggests that the importance of IRGM in the pathogenesis of CD lies in the inhibition of pathogenic bacteria through autophagy activation. However, the relevance of autophagy in CD to IRGM remains controversial, as autophagy activation was observed in Paneth cells of CD patients, whereas CD susceptibility was not associated with IRGM variants. Furthermore, RNA analysis has shown that most autophagy gene sets are downregulated after the appendectomy, thus contributing to the prevention of UC (116). The inhibition of autophagy may provide cross-reactive immunity between host antigens and microorganisms by reducing antigen processing, thereby improving colitis symptoms (116).

### 4.3 NOD2

The NOD2 protein serves as an intracytoplasmic pattern recognition receptor with an essential role in the recognition and defense against the invasion of pathogens and foreign components into the cytoplasm. Its function involves immune response, autophagy, and intracellular bacterial sensing, playing a critical role in host–pathogen interactions and inflammatory responses (117). Antimicrobial autophagy is a major pathway for maintaining homeostasis and mucosal barriers in the living intestine. Several genes at IBD susceptibility loci restrict intracellular bacteria, including ATG16L1, NOD2, IRGM, CALCOCO2/NDP52, and GPR65 (118–121). These genes function in various steps of the antimicrobial autophagic cascade reaction, including bacterial sensing by NOD2, bacterial targeting by IRGM and CALCOCO2, autophagosome extension by ATG16L1, and lysosomal function by GPR65. NOD2 in IBD susceptibility genes acts both as a sensor for the detection of intracellular bacterial peptidoglycan in intestinal epithelial cells and as a direct antimicrobial factor (122).

PaNOD2 overexpression from *Plecoglossus altivelis* can activate the NF-κB signaling pathway, leading to upregulation of TNF-α and IL-1β in HEK293T cells, monocytes, and macrophages (123, 124). The NOD2 gene can affect secretory immunoglobulin A (sIgA) transport in human and mouse M cells by downregulating the expression of two receptors, dendritic cell-associated C-type lectin 1 (dectin-1) and sialic acid-binding Ig-like lectin 5 (Siglec-5), which are involved in retrograde transport. Mutations or deletions in the NOD2 gene can promote the reverse transport of slgA and increase the sIgA–pathogen complex through M cells into intestine-associated lymphoid tissues, leading to the worsening of IBD (125). In addition, the NOD2 gene can also activate the NF-κB pathway by recognizing sites on the intestinal bacterial cell wall and by recruiting NLRP, which acts synergistically with TLR to recognize and bind bacterial lipoproteins, resulting in high expression of pro-inflammatory factors (83). The post-transcriptional regulation mediated by miRNAs has an influential role in autophagy in IBD. Moreover, miRNAs such as miR-320, miR-192, miR-132, and miR-223 can regulate NF-κB or mTOR signaling by targeting the NOD2 gene and releasing pro-inflammatory factors to induce enterocyte autophagy or releasing anti-inflammatory factors to inhibit enterocyte autophagy (84). The activation of the NOD2 gene can reduce the growth of Gram-negative bacteria in gut microbes, attenuate endotoxemia and inflammatory responses, and prevent obesity-induced type 2 diabetes by enhancing the immunoprotective effects of T helper type 1 (Th1) cells (84).

The interaction between NOD2 and ATG16L1 in antimicrobial autophagy persists, and this interaction is thought to be important in facilitating bacterial clearance (81). The process by which NOD2 targets ATG16L1 at the site of bacterial entry into the plasma membrane is a critical step in the elimination of bacteria by autophagy (80). The CD-related shift mutation in NOD2 encodes a mutated NOD2 protein, which then fails to recruit ATG16L1 directionally to the site of bacterial entry into the plasma membrane, resulting in inefficient autophagic clearance of bacteria, which is strongly linked to the production of IBD by NOD2 gene polymorphisms and abnormal expression (126).

It has also been shown that when the NOD2 gene binds to muramyl dipeptide (MDP) in intestinal epithelial cells, it activates NF-κB and mitogen-activated protein kinase (MAPK) signaling pathways to promote autophagic clearance of invading pathogens (85). In contrast, functional NOD2 gene deficiency will lead to a decrease in the secretion of antimicrobial peptides by immune cells, which will affect the encapsulation and phagocytosis of invading pathogens by autophagosomes. The chance of NOD2 gene mutation is the highest risk among all IBD genetic variants, and NOD2 gene mutation leads to a 3- to 20-fold increase in the risk of CD in the organism (127). Therefore, further studies are needed to verify whether suppression of NOD2 gene mutations can be a strategy to improve the condition of IBD.

### 4.4 LRRK2/MUC19

LRRK2/MUC19 is a complex protein containing the GTPase structural domain, C-terminal ROC structural domain, and Ser/Thr kinase structural domain of the RAS of complex proteins (ROC) involved in NOD2-mediated signaling with autophagy as its downstream process (128). LRRK2 is a gene involved in the pathogenesis of Parkinson’s disease, and most early studies were performed in neuronal cells. However, meta-GWASs confirmed the link between LRRK2 and CD and leprosy, and it was hypothesized that LRRK2 acts as an immunomodulator in infection and inflammation. Induced by IFN-γ, LRRK2 is highly expressed in myeloid and B cells and is involved in the production of inflammatory cytokines as well as in the repressive response in macrophages (86). In addition, LRRK2 is required for the cargo sorting process driven by commensal bacteria, by recruiting lysozyme-containing vesicles in Paneth cells, thus participating in coordinating the intestinal lysozyme sorting process to promote commensalism (88).

The molecular mechanisms of how LRRK2 affects CD pathogenesis have not been extensively studied. LRRK2 is a potent negative regulator of the nuclear factor of activated T cells (NFAT), and earlier studies have shown that the lack of LRRK2 negatively regulates the activation of the transcription factor NFAT, leading to increased susceptibility to DSS-induced colitis in mouse models (87). Membrane-associated LRRK2 (associated with TAB2) leads to the inactivation of Beclin-1 and inhibition of autophagy, and both lymphoblastoid cells from control patients carrying a high-risk allele of LRRK2 and dendritic cells from CD patients exhibit elevated LRRK2 expression, leading to increased Dectin-1-mediated NF-κB activation and pro-inflammatory cytokine responses, resulting in more severe colitis (89). LRRK2 is detected in inflamed intestinal tissues, particularly in macrophages of the lamina propria, and functions in host defense by regulating the production of reactive oxygen species (ROS) (86). Collectively, these studies suggest that alterations in LRRK2 are important for the prevention and treatment of colitis and related infections.

### 4.5 ATG7

ATG7 is an E1-like activating enzyme that promotes the formation of autophagic vesicles through two ubiquitin-like binding systems: LC3 lipidation and ATG12 binding. The function of ATG7 was studied in intestinal cells using intestinal epithelium-specific (tamoxifen-inducible) ATG7 KO mice. It was shown that ATG7 KO mice showed defective granular cytostasis in the ileum and Paneth cells. In the small intestinal tissue of ATG7 KO mice, granules were reduced, Paneth cell lysozyme levels were decreased, and the expression of TNF-α and IL-1β mRNA was increased (90). Further studies have shown that the exacerbation of colitis symptoms in ATG7 conditional knockout (cKO) mice is associated with abnormal bacterial flora composition, dysregulated expression of antimicrobial peptides, and inhibition of colonic mucin secretion (92).

Autophagy plays a role in maintaining intestinal homeostasis in general control non-derepressible 2 (GCN2)^−/−^ CD11c^+^ antigen-presenting cells (APCs) or intestinal epithelial cells, and conditional ablation of Atg7 in intestinal APCs resulted in enhanced ROS and Th17 responses (93). Not only that, mice with myeloid-specific deletion of ATG7 had increased susceptibility to colitis and more severe symptoms (129). Furthermore, ATG7 deficiency in intestinal epithelium-specific XBP1-deficient mice exacerbated intestinal lesions, leading to submucosal inflammation or wall-piercing inflammation, suggesting that autophagy is involved in the compensatory process of intestinal epithelium during ongoing endoplasmic reticulum stress (130). These studies suggest that ATG7 has an important role in regulating the intestinal inflammatory response and defending against intestinal pathogens to maintain intestinal homeostasis. However, there is little evidence that ATG7 has clinical relevance in IBD.

### 4.6 MTMR3

Myotubularin-related protein 3 (MTMR3) is an important member of the myotubular-related protein family (131). It shares extensive homology with myotubularin, including the catalytic structural domain, but additionally has a C-terminal extension containing the FYVE structural domain (132). MTMR3 is an inositol lipid phosphatase that is present in both the cell membrane and cytoplasm (133). In terms of biological function, MTMR3 is an important component of autophagy (134), determining the initiation of cellular autophagy and the size of the membrane structure of the autophagosome by regulating phosphatidylinositol 3-phosphate (PtdIns3P) (94). MTMR3 is a cellular autophagy-related gene and is associated with the risk of rheumatoid arthritis and systemic lupus erythematosus (96). Therefore, MTMR3 is biologically involved in cellular autophagy, cell migration, and invasion and is closely related to cancer cell growth and development.

The role of MTMR3 in regulating the PRR associated with the intestinal immune system is poorly studied. MTMR3 exerted an influential role in PRR initiation response in primary human macrophages, and in response to PRR stimulation, MTMR3 decreased Ptd Ins3P levels and autophagy. In turn, it also increases caspase-1 activation and autocrine IL-1β secretion, PI3K, and NF-κB activation, elucidating the mechanism by which MTMR3 regulates PRR-induced autophagy as well as cytokine secretion, a process critical in the regulation of intestinal immune homeostasis (95).

### 4.7 HMGB1

The high mobility group box 1 protein (HMGB1) is a DNA-binding protein that is widely found in various mammalian species. In the nucleus, HMGB1 is involved in the construction and stabilization of nucleosomes as well as the regulation of gene transcription. It has been found that HMGB1 has both pro-inflammatory and pro-tissue repair roles in the extracellular domain, and therefore, its unique role in inflammation and tissue repair is of great interest. The intracellular protein HMGB1 is released from cells and is present in many diseases as an injury-related molecule. It was found that extracellular HMGB1 promotes inflammation, while intracellular HMGB1 effectively has an anti-inflammatory effect, mainly through the ability of HMGB1 to protect Beclin-1 and ATG5, reduce apoptosis and thus tissue inflammation, prevent the release of intracellular inflammatory mediators, and maintain tissue barrier integrity. HMGB1 can also determine whether apoptosis or autophagy occurs during calpain activation (97). Therefore, it can be used as a new target for human disease treatment.

### 4.8 Others

PTPN2-deficient macrophages can express high levels of CD86 and increase IL-6 secretion, increasing the likelihood of IBD. This gene-deficient macrophage preferentially differentiates into pro-inflammatory macrophages, increasing damage to the intestinal epithelial barrier (135). PTPN2 deficiency impairs the bacterial killing function of granulocytes, making it easier for the bacteria to translocate beyond the intestinal epithelial barrier, thus making the gut more susceptible to inflammatory responses (136). In CD, PTPN2 deficiency alters the infiltration of various immune cells in the intestinal lamina propria, with a marked increase in macrophages. Hering et al. suggested that the organism may compensate for the loss of PTPN2-mediated anti-inflammatory response by an increase in macrophages, thus avoiding an increase in the severity of intestinal inflammation (137).

ATG4B is a critical protein for the formation and maturation of autophagosomes and can provide cells with sufficient LC3 to enhance autophagic flux. ATG4B is able to recycle lipidated LC3 to maintain autophagy, and loss of function leads to sensitivity of cancer cells to chemotherapy and radiotherapy (138). Therefore, the development of inhibitors targeting ATG4B can realize the inhibition of autophagy.

In addition, interleukin-23 receptor (IL-23R), X-linked inhibitor of apoptosis (XIAP), ULK1, and vitamin D receptor (VDR) are all autophagy gene variants and proteins associated with IBD (139).

The exact mechanisms underlying the pathogenesis of IBD are not yet fully characterized, and the large number of genetic loci explains only a minor portion of the variance in the disease (140). However, genetic studies still play a crucial role in the diagnosis and treatment of IBD. The discovery of susceptibility genes provides an avenue for disease risk prediction, and by implementing early screening, we can intervene in the early stages of disease progression, thereby reducing the risk of missing treatment in susceptible populations. For high-risk groups with a family genetic history, early intervention is recommended to reduce the likelihood of disease development. The study of susceptibility genes helps to provide accurate disease typing and risk assessment of patients, thus guiding the selection of targeted therapeutic drugs and realizing personalized medicine. These susceptibility genes also provide important tools for assessing disease severity and prognosis. The study of susceptibility genes not only helps to understand the pathogenesis of IBD but may also reveal new therapeutic targets for the development of new therapeutic approaches.

## 5 Autophagic modulation strategies in IBD

Autophagy can remove aggregated proteins, damaged organelles, and infectious pathogens, which is important for maintaining normal cellular homeostasis and energy balance. Autophagy disorders are associated with the development of various diseases. Many autophagy modulators are excellent tools for studying and validating the therapeutic significance of autophagy because of their accessibility, rapid effect, and reversible regulation. The above genetic variation studies provide a molecular basis for the development of therapeutic strategies targeting autophagy. Many autophagy modulators have been identified in the current treatment of IBD, but most have not been applied in clinical practice (**Table 2**). Most of these reported compounds suffer from aspects such as low specificity or low potency, and therefore, the use of a variety of autophagy modulators with different modes of action is needed to achieve accurate studies of the autophagic process (154). The development of regulatory strategies that act highly selectively on the autophagic process is one of the major challenges in this field. Not only that, there are fewer drug screening methods for the discovery of autophagy modulators, so the field is in dire need of new technological approaches to facilitate the discovery of autophagy modulators with more selective and therapeutic potency. Therefore, in addition to some autophagy regulators in **Table 2**, several bioprobe strategies have been developed to help researchers better understand the biological mechanisms of diseases by modulating specific targets or pathways.

**Table 2:** Pharmacological applications of autophagy regulators in the treatment of IBD.

Autophagy regulators	Pharmacological mechanisms	Reference
GL-V9	Activates AMPK signaling and degrades the NLRP3 inflammasome complex in macrophages	Zhao et al. (141)
Ginsenoside Rd	Mediates NLRP3 inflammasome inactivation via induction of P62-driven mitochondrial autophagy	Liu et al. (142)
Palmatine	Promotes mitochondrial autophagy and inhibits NLRP3 inflammasome	Mai et al. (143)
Evodiamine	Inhibits NLRP3 inflammasome assembly	Ding et al. (144)
Metformin+MCC950	Modulates the interaction of HSP90 with NLRP3 and inhibits autophagy-mediated NLRP3 inflammasome	Saber and El-Kader (145)
Kynurenic acid	Induces autophagy-dependent degradation of NLRP3 via the GPR35 axis	Zheng et al. (146)
HU308	Regulates the AMPK-mTOR-p70S6K signaling pathway and inhibits NLRP3 inflammasome activation	Ke et al. (147)
Nicotine	Regulates the AMPK-mTOR-P70S6K signaling pathway	Gao et al. (148)
PNU282987	Mediates autophagy by inducing AMPK-mTOR-p70S6K signaling	Shao et al. (149)
PCA1	Promotes autophagy via the AMPK/mTOR/p70S6K signaling pathway	Zhang et al. (150)
Galangin	Promotes autophagy-mediated benign regulation of gut microbes	Xuan et al. (151)
LR12	Inhibits TREM-1 and promotes the expression of autophagy and CMA-related proteins	Parent et al. (152) Kokten et al. (153)

### 5.1 lncRNA

lncRNA, or long non-coding RNA, are non-coding transcripts exceeding 200 nucleotides in length, predominantly localized within the cell nucleus. Among these, lncRNA-H19 exhibits high expression in the colon of UC mice. Li et al. (155) demonstrated that lncRNA-H19 acts as a molecular decoy, sequestering miR-331-3p to inhibit its activity. This suppression promotes the transcription of tumor necrosis factor receptor-associated factor 4 (TRAF4), ultimately inducing cellular apoptosis. Xu et al. (156) reported that exosomes derived from human umbilical cord mesenchymal stem cells overexpressing lnc78583 alleviate lipopolysaccharide-induced inflammation in human colon epithelial cells. This effect is mediated via the lnc78583/miR-3202/HOXB13 axis, potentially involving autophagy inhibition. Furthermore, overexpression of lncRNA-PMS2L2 promotes miR-24 methylation, indirectly mitigating the pro-apoptotic effects of miR-24 in UC cells (157). lncRNA-TUG1 functions as a molecular sponge, regulating the balance between the RNA-binding protein HuR (human antigen R) and miR-29b-3p to inhibit apoptosis in intestinal epithelial cells during colitis (158). Similarly, lncRNA-SNHG5 acts as a scaffold, modulating the miR-375/JAK2 axis to promote proliferation and suppress apoptosis in adult mouse colon cells (159).

### 5.2 NLRP3 inflammasome

**Figure 3:**
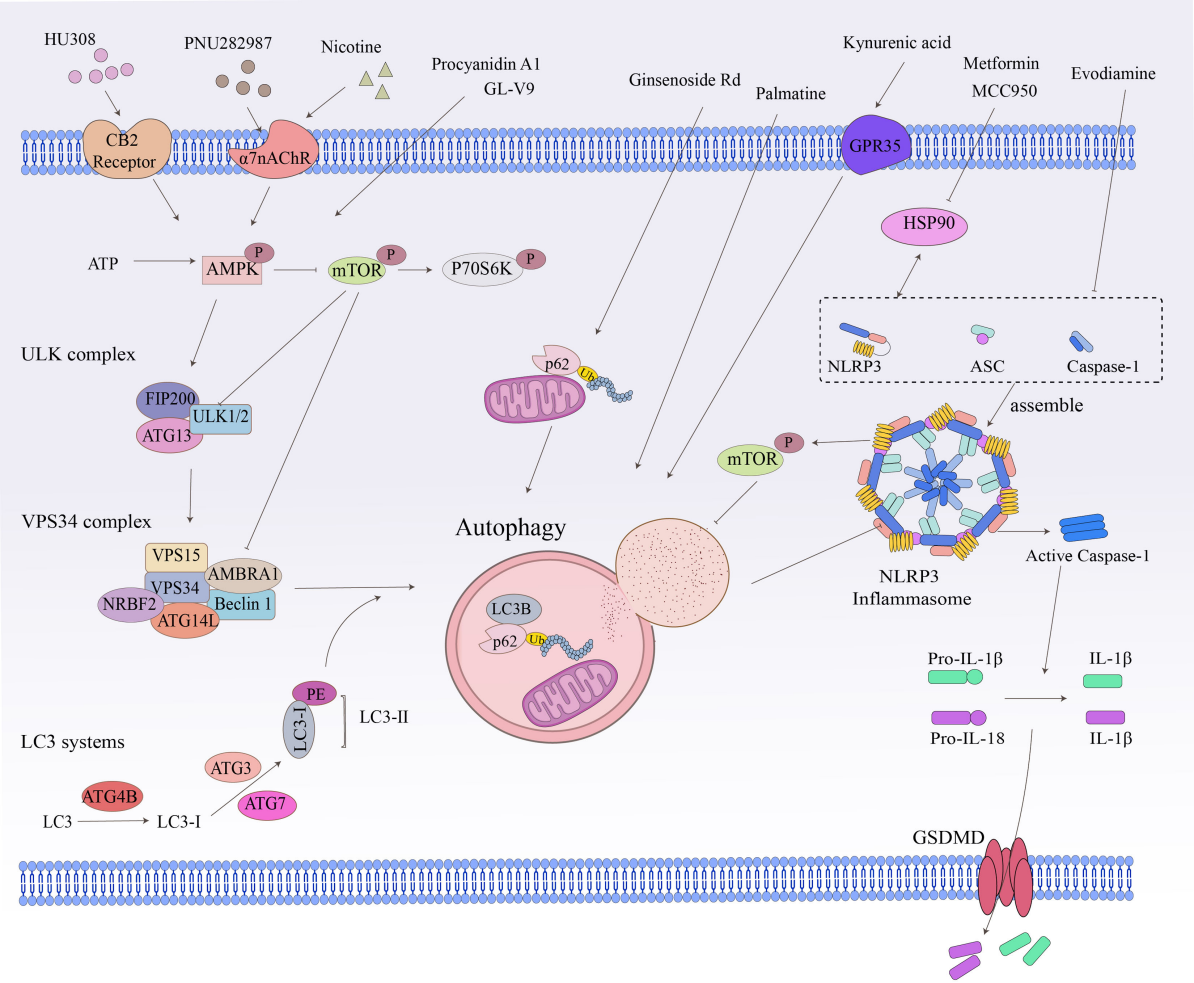
Crosstalk between related signaling pathways and autophagy. Cellular autophagy involves numerous intracellular signaling pathways and the degradation and recycling of biomolecules and damaged organelles. The ATP, ULK complex, VPS34 complex, LC3-II, and P62 are all hallmark proteins of autophagy. The mTOR kinase is a critical molecule in the induction of autophagy. Pathways that activate mTOR, such as NLRP3 and apoptosis signaling, inhibit autophagy, while pathways that negatively regulate mTOR, such as the AMPK signaling pathway, promote autophagy.

Inflammasomes are multiprotein oligomers responsible for the activation of the inflammatory response, which belongs to the innate immune family and is mainly found in epithelial cells and most inflammatory, immune cells, such as macrophages and dendritic cells in the intestine (160, 161). The signaling crosstalk between the inflammasome and autophagy has been well-investigated in many diseases (**Figure 3**) (162). To date, several members of the autophagy-related inflammasome have been described, including NLRP1, NLRP3, the NLR family caspase recruitment domain-containing protein 4 (NLRC4), and double-stranded DNA sensors absent in melanoma 2 (AIM2).

Signaling crosstalk between non-NLRP3 inflammasome (e.g., NLRP1, NLRC4, AIM2) and autophagy is rarely reported in inflammatory diseases (163). NLRP3, unlike other inflammasomes, can be activated by a broader range of stimuli and is therefore the most studied and well-characterized inflammasome. The mechanism of NLRP3-mediated autophagy regulation and its relation to inflammatory diseases has been progressively elucidated (164), especially the discovery that the autophagy-associated protein IRGM regulates autophagy in cells by inhibiting NLRP3 (75). NLRP3 is a binding chaperone of the autophagy inhibitor mTOR (165). During inflammatory conditions, NLRP3 inflammasome binds and promotes mTOR phosphorylation, which inhibits autophagy and disrupts autophagy-mediated elimination of pro-inflammatory mediators, thereby exacerbating inflammation (165).

The crosstalk between NLRP3 and autophagy performs an essential role in innate immunity against bacterial, fungal, and viral infections (166) and has been widely reported to be associated with the pathogenesis and progression of IBD (167). In DSS-induced IBD mice or *Il10* ^−/−^ mice, hypoxia suppresses intestinal inflammation by downregulating NLRP3-mTOR binding, thereby activating autophagy-mediated degradation of NF-κB signaling mediators and decreasing the expression of pro-inflammatory genes (165). In response to intracellular pathogens, caspase-4 is activated, leading to inflammasome activation, which positively regulates macrophage autophagic vesicle biogenesis and translocation to lysosomes, with increasing hetero-autophagy-mediated elimination of pathogens (168). Thus, excessive activation of the NLRP3 inflammasome has a significant impact on the development and progression of IBD.

The inhibition of NLRP3 inflammasome activation is a potential therapy for inflammatory diseases (166). Some autophagy modulators alleviate IBD by inhibiting the activation of NLRP3 inflammasome (144, 146). GL-V9, an agonist of the small molecule adenosine 5′-monophosphate-activated protein kinase (AMPK), significantly degrades the NLRP3 inflammasome complex in macrophages by inducing autophagy, thereby preventing colitis (141). The tetracyclic triterpene derivative ginsenoside Rd mediates NLRP3 inflammasome inactivation via induction of P62-driven mitochondrial autophagy, which significantly attenuates the severity of DSS-induced colitis in UC mice (142). The natural derivative Palmatine ameliorated DSS-induced colitis by promoting mitochondrial autophagy-mediated NLRP3 inflammasome inhibition (143). Evodiamine, a natural product, inhibits NLRP3 inflammasome assembly to activate cellular autophagy, attenuates experimental DSS-induced colitis injury, and suppresses NLRP3 inflammasome by inhibiting apoptosis-associated speckle-like protein oligomerization and caspase-1 activity in macrophages (144). MCC950 is a small molecule NLRP3 inflammasome inhibitor that reduces the production of pro-inflammatory cytokines such as IL-1β and IL-18 (169). In addition, the combination of metformin and MCC950 produced a palliative effect on UC, a strategy that attenuates DSS-induced colitis by modulating the interaction of heat shock protein 90 (HSP90) with NLRP3 and inhibiting autophagy-mediated NLRP3 (145). Kynurenic acid, a colitis-associated endogenous regulator, induces autophagy-dependent degradation of NLRP3 in macrophages via the kynurenic acid/G-protein-coupled receptor 35 (GPR35) axis (146).

### 5.3 AMPK

In the immune microenvironment, AMPK and mTOR are important molecules involved in the regulation of cellular autophagy, and activated AMPK directly promotes autophagy by phosphorylating autophagy-related proteins in mTORC1, ULK1, and PIK3C3/VPS34 complexes or indirectly promotes autophagy by regulating the expression of autophagy-related genes downstream of transcription factors (106). The AMPK-mTOR-p70 ribosomal protein S6 kinase (p70S6K) pathway is a classic pathway in the activation of autophagy and mediates the pro-autophagic effects of various targets other than the inflammasome (**Figure 3**) (147, 170). Cannabinoid receptor 2 (CB2R), a member of the G-protein-coupled receptor (GPCR) family, is increasingly being studied as an immune and inflammatory modulator (18). In contrast to CB1R, which is predominantly expressed in the central nervous system, CB2R is primarily located in the immune system, including macrophages and other inflammatory and immune cells in peripheral tissues (18). The administration of CB2R agonist HU308 activation inhibits NLRP3 inflammasome activation and induces autophagy activation in intestinal macrophages to reduce the severity of DSS-induced colitis in mice, and the protective effect on IBD was mediated by the autophagy-related pathway AMPK-mTOR-p70S6K signaling (147). The alpha7 nicotinic acetylcholine receptor (α7nAChR) is a member of the “Cys-loop” cationic ligand-gated channel superfamily. It has been shown to act by triggering the “cholinergic anti-inflammatory pathway” (170). It has been reported that α7nAChR knockout largely aggravated DSS-induced colitis and the severity of IBD in mouse BMDM stimulated by LPS/DSS (149). Nicotine, a non-selective α7 nicotinic acetylcholine receptor (α7nAChR) agonist, ameliorates DSS-induced colitis *in vivo* and *in vitro* by activating the AMPK-mTOR-p70S6K signaling pathway. This protective effect was significantly attenuated by the autophagy inhibitor 3-methyladenine (3-MA), suggesting an autophagy-dependent mechanism (148). PNU282987 is another selective α7nAChR agonist that mediates autophagy by inducing AMPK-mTOR-p70S6K signaling in intestinal macrophages to resist DSS-induced colitis (170). Procyanidin A1 (PCA1) is a kind of procyanidin. Recent research has demonstrated that PCA1 treatment alleviated DSS-induced UC by increasing the expression of p-AMPKα and inhibiting the levels of its downstream pathways p-mTOR and p-p70S6K. In addition, in LPS-induced HT-6 cells, with the combined application of PCA1 and the AMPK antagonist compound C, the expression of Beclin-1 and the LC3-II/I ratio was inhibited, and the level of p62 was increased. These data suggested that PCA1 promoted autophagy in UC via the AMPK/mTOR/p70S6K signaling pathway (150).

### 5.4 Gut microbial modulators

Disturbances in gut microbial homeostasis are closely associated with the development of IBD, and restoring this homeostasis is a potential therapeutic strategy for IBD (171). Autophagy has been revealed to play an essential role in gut regulation, contributing to the regulation of the gut microbiota in IBD. Therefore, autophagy-mediated regulation of the gut microbiota can be exploited to alleviate IBD (172). Among these, mutations in autophagy-associated ATG16L1 and NOD2 lead to the inactivation of atypical autophagic processes, resulting in dysbiosis of the intestinal flora in IBD (173).

Vitamin D promotes intestinal autophagy and attenuates IBD, and vitamin D/VDR signaling is beneficial in maintaining and restoring gut microbiota homeostasis (174). The natural flavonoid galangin may be used to treat DSS-induced colitis by promoting autophagy-mediated benign regulation of gut microbes in small intestinal epithelial cells (151). The triggering receptor expressed on myeloid cells 1 (TREM-1) is expressed on most innate immune cells, but less on parenchymal cells. In biopsies from UC and CD patients, the proportion of neutrophils and macrophages with high TREM-1 expression was significantly higher in inflammatory biopsies than in non-inflammatory biopsies (175). The inhibition of TREM-1 contributed to the restoration of impaired autophagic activity in neutrophils and macrophages, thereby positively regulating the gut microbes of IBD mice (153). The peptide LR12 was shown to inhibit TREM-1 in studies and to alleviate colitis in DSS-induced mice at clinical signs and endoscopic and histological levels (152). The expression of autophagy (ATG1, ATG13, ATG5, and ATG16L1) and CMA (HSPA8 and HSP90AA1)-related proteins was significantly increased after LR12 injection in DSS-induced mice, and this effect was also confirmed by experiments using Trem-1 KO mice (153).

### 5.5 Others

The selective autophagy receptor optineurin is also considered to be a critical factor in maintaining pathogen clearance and regulating macrophage cytokine production (176). Optineurin mediates macrophage autophagy, thereby inhibiting the intestinal macrophage-mediated inflammatory response and helping to reduce mucosal damage in IBD, which is a potential target for IBD treatment (177).

In addition, the latest studies have shown that autophagy and extracellular vehicles (EVs) synergistically regulate IBD through a bidirectional interaction network. Autophagy defects drive the secretion of inflammatory EVs. When the autophagy pathway of intestinal epithelial cells (such as the ATG16L1/ULK1 complex) is damaged, the abnormally accumulated damaged mitochondria are packaged into EVs through the PINK1/Parkin-independent pathway, goblet cells are defective in autophagy, and the integrity of the mucus barrier is destroyed (178). EVs can also inhibit pro-inflammatory cytokines, promote anti-inflammatory M2 macrophage polarization, promote tissue repair, and restore intestinal homeostasis. The core mechanism of this autophagy–EV–IBD regulatory axis identifies the challenges and opportunities associated with its translation into clinical practice (179).

## 6 Clinical translation of autophagy modulators

The main therapeutic drugs for IBD in clinical practice are aminosalicylic acid drugs, corticosteroids, and immunosuppressants, as well as the use of biological agents, which are mainly used to alleviate the symptoms and control the development of the disease (180). However, some of the drugs have many adverse effects, and some patients do not respond to the above drug regimens and cannot be treated continuously, resulting in poor therapeutic effects and causing great economic and psychological burdens to patients and their families. Therefore, the treatment of IBD is still a problem that needs to be explored for more solutions. Increasing evidence supporting the important role of autophagy in maintaining intestinal homeostasis and protecting the intestinal mucosal barrier has raised enthusiasm for targeting autophagy to improve IBD, and there is great potential for significant advances in the future in the diagnosis and treatment of IBD through the restoration of impaired autophagy and the specific modulation of autophagy. At present, the research on autophagy modulators is still at the cellular and animal level, and no mature clinical trials have been reported. However, based on the available animal and clinical evidence, autophagy modulation strategies could be considered as a potential therapeutic approach for IBD. The absence of key autophagy-related genes increases susceptibility to IBD (69, 181); in contrast, autophagy dysfunction can mediate IBD-associated intestinal mucosal barrier disruption (25, 182, 183). Furthermore, there is a certain link between autophagy and almost all inflammatory pathways of cells (181). Autophagy modulation strategies can help the body correct the imbalance process and compensate for the deficiencies of existing biological agents. Some inhibitors are small molecule modulators or small peptides that circumvent immunogenicity and are suitable for patients who are intolerant to biomolecular agents or resistant to antibody therapy. For the better clinical translation of autophagy modulation strategies, the support of more clinical trials is now needed, to assess the unintended consequences or adverse effects of therapeutic strategies, to utilize extended studies of autophagy modulators in TNF-α antibody-resistant IBD, and to accurately monitor available biomarkers of mucosal healing in IBD patients (184).

Despite significant advances in elucidating the autophagy–IBD axis, translating these insights into clinical practice remains a formidable challenge. A persistent gap exists between mechanistic discoveries and their implementation in patient care. One of the primary obstacles is that most findings related to autophagy are derived from animal models or *in-vitro* systems, with limited validation in human cohorts. This lack of translational evidence significantly impedes the clinical utility of such research. In IBD therapeutics specifically, although numerous autophagy-related targets have been identified, only a handful have progressed to clinical trials, and even fewer have led to approved interventions—underscoring a critical bench-to-bedside disconnect.

Compounding this challenge is the intrinsic complexity of autophagy itself. Its highly context-dependent and cell-type-specific roles complicate the development of targeted therapies. Current strategies to modulate autophagy often lack precision, raising concerns about off-target effects and safety. For instance, indiscriminate activation or inhibition of autophagy may provoke divergent or even contradictory responses across different intestinal cell populations, resulting in unpredictable clinical outcomes.

To overcome these translational hurdles, future research must adopt clinically oriented approaches, including leveraging patient-derived multi-omics datasets to identify individualized and disease-relevant autophagy targets, developing precision therapeutics capable of selectively modulating autophagy in specific cell types or pathological contexts, and establishing predictive preclinical models that better reflect human disease heterogeneity and therapeutic responses.

In summary, while our understanding of autophagy in IBD pathogenesis continues to expand, its clinical translation remains in its infancy. Bridging this gap will require concerted efforts that integrate basic science with translational research, ultimately enabling the development of effective, targeted therapies for IBD patients.

## 7 Deficiencies and prospects

Despite growing recognition of autophagy’s pivotal role in IBD pathogenesis, its clinical translation faces significant constraints. The first thing is the context-dependent duality. Autophagy exhibits cell-type- and disease-stage-specific functionality (such as intestinal epithelial cells, Paneth cells, and macrophages), resulting in opposing effects that challenge broad therapeutic modulation (185, 186). While autophagy-related gene variants (e.g., ATG16L1, NOD2, IRGM) confer IBD susceptibility, their variable penetrance and divergent functional impacts across individuals complicate target selection and limit therapeutic generalizability. Note the pharmacological imprecision. Current autophagy modulators act non-specifically, disrupting multiple pathways and incurring risks of off-target effects, immunosuppression, and compromised host defense—particularly detrimental in IBD with pre-existing barrier dysfunction and dysbiosis. Furthermore, the absence of reliable *in-vivo* autophagy activity biomarkers impedes patient stratification and real-time therapeutic response monitoring.

Although autophagy remains a promising IBD target, its therapeutic exploitation demands the refined dissection of cell-specific mechanisms, next-generation agents with spatiotemporal precision, and the translational tools to bridge mechanistic insights and clinical deployment.

## 8 Conclusion

The pathogenesis of IBD as a non-specific intestinal inflammatory disease is complex, and the specific etiology and pathogenesis are not yet fully defined. It has been shown that autophagy and its related genes are involved in the immune response of IBD through various pathways such as pathogen clearance, immune function, regulation of inflammatory signaling, or inhibition of inflammasomes. The main role of therapeutic drugs for IBD in clinical practice is to relieve symptoms and control the progression of the disease (187). However, some of the drugs have many adverse reactions. A few patients do not respond to the above drug regimens and are unable to sustain treatment, which is less effective and imposes a great financial and psychological burden on patients and their families. Therefore, the treatment of IBD remains a problem for which more solutions need to be explored.

Autophagy is an important process for the body to maintain cellular metabolic homeostasis and endostasis, which is involved in the regulation and function of multiple signaling pathways in the body. Autophagy is involved in maintaining intracellular homeostasis and is closely related to the repair of intestinal mucosal barrier damage, innate immune response, adaptive immune response, and defense against pathogenic microorganisms. With the discovery of autophagy-related genes, rapid progress has been made in understanding the biology of autophagy and its relevance to the health and disease of organisms (188). Autophagy has become a hotspot in recent years in the pathogenesis and clinical diagnosis and treatment of IBD and other diseases due to its modulation characteristics. The recent research progress has fully revealed the important correlation between autophagy abnormalities and IBD, suggesting that modulation of autophagy abnormalities is expected to be a new strategy for IBD prevention and treatment. Currently, there are three classes of extensively studied autophagy modulators as candidates for IBD treatment, including inflammasome inhibitors, gut microbiota modulators, and AMPK-mTOR-p70S6K signaling modulators, which greatly expand the new horizon of autophagy modulation strategies applied to IBD treatment and open up the space for the development of new therapeutic strategies. Targeted intervention on autophagy-related molecular mechanisms can effectively block the disease progression of experimental UC and CD. However, our understanding of the molecular mechanisms underlying the multiple functions of autophagy proteins in immune-related processes is still rather primitive, and current proteomic and genomic screens have the potential to transform autophagy and immune studies, lacking studies of the pathogenic mechanisms of IBD through the influence of autophagy genes on specific pathways. An in-depth understanding of the mechanism of autophagy in IBD and the effectiveness of targeted therapy will provide new targets and new ideas for clinical disease prevention and treatment.

Despite the increasing number of studies on the mechanisms of autophagy regulation in intestinal cells in recent years, few autophagy modulators have been successfully applied in clinical practice. Because of the complexity of autophagy regulation in IBD, further research is needed to achieve the ultimate application of autophagy therapeutic strategies in the clinical setting. It is expected that with further studies on more autophagy-related genes, the autophagic pathways that influence the development of IBD can be identified and provide meaningful targets for clinical treatment. The research to explore the mechanism of autophagy in IBD can provide new insights regarding the treatment of patients. However, to promote the translation of basic research into clinical treatment, there are still many issues that need to be elucidated, such as the dynamics of autophagy abnormalities in the development of IBD, the key signaling molecules that promote and inhibit autophagy in the intestinal microenvironment, and the differential regulation of autophagy in IBD by different cell subtypes, which can help to identify the targets for drug regulation and design precise therapeutic regimens.
